# Animations in Cross-Platform Mobile Applications: An Evaluation of Tools, Metrics and Performance

**DOI:** 10.3390/s19092081

**Published:** 2019-05-05

**Authors:** Andreas Biørn-Hansen, Tor-Morten Grønli, Gheorghita Ghinea

**Affiliations:** 1 Department of Technology, Kristiania University College, 0670 Oslo, Norway; tor-morten.gronli@kristiania.no; 2 Department of Computer Science, Brunel University, London UB8 3PH, UK; george.ghinea@brunel.ac.uk

**Keywords:** cross-platform mobile development, app development, app animations, user interfaces

## Abstract

Along with the proliferation of high-end and performant mobile devices, we find that the inclusion of visually animated user interfaces are commonplace, but that research on their performance is scarce. Thus, for this study, eight mobile apps have been developed for scrutiny and assessment to report on the device hardware impact and penalties caused by transitions and animations, with an emphasis on apps generated using cross-platform development frameworks. The tasks we employ for animation performance measuring, are those of (i) a complex animation consisting of multiple elements, (ii) the opening sequence of a side menu navigation pattern, and (iii) a transition animation during in-app page navigation. We employ multiple performance profiling tools, and scrutinize metrics including frames per second (FPS), CPU usage, device memory usage and GPU memory usage, all to uncover the impact caused by executing transitions and animations. We uncover important differences in device hardware utilization during animations across the different cross-platform technologies employed. Additionally, Android and iOS are found to differ greatly in terms of memory consumption, CPU usage and rendered FPS, a discrepancy that is true for both the native and cross-platform apps. The findings we report are indeed factors contributing to the complexity of app development.

## 1. Introduction

Nowadays, we frequently encounter animated graphical user interfaces in mobile applications  [1], or apps for short. Use cases typically include those of branding purposes, communication of information and interaction patterns, training, and to enhance overall user experience [2]. Throughout a regular day’s worth of mobile device usage, we are likely to have been exposed to a multitude of different animations. Examples include the loading spinners appearing upon refreshing social media feeds, the animated proposed travel path drawn by the Uber app on-top of their Google Map component, and the view-filling animation transition triggered when launching an app on an iOS device [3]. Thus, it should be safe to assume that animations and transitions are integral parts of modern user interfaces, also on performance- and view-estate constrained handheld mobile devices.

Not only do app developers face such inherent device-related constraints, they are also exposed to a myriad of technological options to choose between prior to even beginning development. In mobile app development, traditionally, apps have been developed using platform-specific languages, Objective-C or Swift for iOS [4], Java [5] or Kotlin [6] for Android, and C# or C++ for the Windows Phone/Mobile/Universal platform [7]. All of these languages add to the list of required competency for developing mobile apps for a widest possible audience, a customer base which in 2018 generated approximately $419 billion USD in revenue, according to recent numbers from Statista [8]. These types of platform-specific apps are referred to as native apps [9], as they are pieces of software written using tools and technologies that are native to their respective platform. In fact, not only do the mentioned platforms require different programming languages, there is also a complete lack of interoperability between them, resulting in developers having to implement all business logic and user interface code from scratch for each platform deemed necessary to have presence on [10]. Ahmad et al. [10] identified this lack of code re-use to be a concern among native app developers, in their empirical study on challenges in mobile development. In addition to challenges concerning code re-use across platforms, the device fragmentation on Android can also render native development a complicated endeavour. Taking into account OpenSignal’s [11] latest report (2015) on Android fragmentation identifying more than 24,000 distinct Android devices, and that Android’s market share in 2017 was 85.9% according to Statista [12], it should be safe to assume that the majority of practitioners develop mobile applications for both a device- and platform-fragmented audience.

To reach this fragmented customer base without investing in platform-specific re-implementations of the same features and user interfaces, cross-platform development emerge to help maximize code re-use across mobile platforms [13]. In our context, *cross-platform* is an umbrella term describing numerous concepts and technical solutions for minimizing the amount of code needed to deploy a codebase to as many mobile platforms as possible. Additionally, a frequently encountered requirement in the literature is that such apps should perform similar to native apps, as discussed in Xanthopoulos and Xinogalos’ well-cited early work on the subject, stating that *"The ultimate goal of cross-platform mobile app development is to achieve native app performance and run on as many platforms as possible."* [14]. Native-like performance is typically tried achieved through the use of cross-platform development frameworks, examples being those of Ionic Framework, Xamarin, React Native [15] and similar tools [5,16]. We categorize these frameworks into approaches depending on characteristics, typically those of execution environment, user interface rendering techniques, and code compiling and transpilation steps [17].

In this study, we employ frameworks of the Hybrid, Interpreted and Cross-Compiled approaches. While they can all aid in the development of cross-platform apps, they differ in terms of characteristics such as those previously mentioned. The Hybrid approach offers the possibility to develop apps using traditional web technologies. The code, typically JavaScript, CSS and HTML, is wrapped inside a regular native app displaying the code through an embeddable web browser component, referred to as a *WebView*. This WebView component can then communicate with platform-specific code such as Java or Objective-C existing in the native app in which the component and web code is wrapped, enabling the use of device- and platform functionality not available to web apps executed in a regular web browser.

Like Hybrid apps, the Interpreted approach relies on an underlying native application to execute the cross-platform code. However, a major difference between the two approaches is that Interpreted apps are not dependent on a WebView component to render user interfaces or communicate with device- and platform functionality. Instead, the Interpreted approach is based on programming language interpreters, including but not limited to JavaScriptCore and V8, to facilitate the rendering of native user interface components along with communication with native code. No web-based components are rendered to the screen when developing Interpreted apps, instead frameworks of the approach can communicate with- and render to the screen components that are as native as those available in traditional native apps.

The final approach included in this study is the Cross-Compiled approach. This allows for the use of a language such as C# to be compiled to platform-specific byte code, and thus avoid the use of interpreters and WebView components all together. Due to executing as native byte code, also the Cross-Compiled approach renders native user interfaces, similar to the Interpreted approach, and can communicate with device and platform features without the need of an abstraction layer such as a WebView or code interpreter [16].

The user experience provided by apps developed using these cross-platform approaches has not been researched to any great extent, as we further discuss in Section 2. The importance of this topic however has been covered in numerous studies, recently by Ahmad et al. [10] and Biørn-Hansen et al. [18,19]. All three studies indicate that user experience is a concern and challenge developers typically relate to cross-platform mobile apps, more so than for native apps [10]. The same studies also indicate that there is a lack of academic effort put forth to investigate the validity of these concerns. For this current study, we are motivated by the identified gap in the knowledge base, along with industry relevance and the potential practical and theoretical implications of our findings. We target specifically animations and transitions in mobile user interfaces, and make use of multiple performance profiling tools and performance-related metrics to discuss and report on animations performance in cross-platform and native apps running on Android and iOS devices.

The rest of this paper is structured as follows: In Section 2, we discuss the state of research on cross-platform development, with a particular focus on performance-oriented studies. Section 3 outlines our research questions and overall design of the study and experiment. An overview of the artifacts designed and developed is presented in Section 4, before we present our results in Section 5, and discuss the experiment and its outcomes in Section 6. A conclusion along with thoughts on further work is then presented in Section 7.

## 2. Related Work

We have identified several studies reporting on the performance of mobile apps generated using cross-platform development approaches and frameworks. However, few of these studies focus on aspects related to user experience. A recent study by Willocx et al. [20] assesses the device impact of cross-platform apps, measuring metrics including CPU, memory (RAM), disk space and time-to-task completion (e.g., launch time and elapsed navigation time) in the context of hardware utilization in different states, including background versus foreground consumption, consumption at launch, and more. We extend their experiment by introducing additional measurement parameters, and by targeting the user interface context rather than performance testing of apps in different states (e.g., foreground and background). Their results illustrate a performance penalty introduced when making use of cross-platform frameworks.

A variety of these metrics are also found included in research of similar nature, such as in Dalmasso et al.’s [21] study evaluating cross-platform frameworks such metrics as CPU, RAM usage and power consumption. Their research is frequently cited in newer studies, possibly due to their empirical approach to evaluation of four technical implementations. The authors find that the Hybrid approach and the PhoneGap development framework performs better in terms of hardware utilization when compared to the Titanium framework of the Interpreted approach. No native implementations were part of the research design. Additionally, the authors provide a set of guidelines for deciding on the suitability of cross-platform approach and framework, in which they express their thoughts on the importance of a native user experience.

In [16], Ciman and Gaggi deviate from the trend of drawing conclusions based on CPU and memory consumption, focusing instead on device energy consumption while executing and performing a variety of tasks involving the use of hardware features. Similar to most research on cross-platform development, also this study can report of a performance penalty introduced by the use of cross-platform frameworks. While we do not extend their work in the direction of investigating power consumption, we do draw on ideas and findings presented throughout their study. An example of this is their finding related to the battery-oriented expense of updating an app’s user interface, which they identify to be the main cause of an increase in energy consumption. Thus, the research they present is of great importance to understand the consequences of unoptimized animations and transitions, as these actions are after all major causes to rapid re-drawing and updating of the user interface.

One study covering the possibilities of animations in cross-platform apps has been identified, being that of Ciman et al. [22]. They investigate the availability of animation API support in cross-platform frameworks, specifically MoSync (https://github.com/MoSync/MoSync), Titanium (https://www.appcelerator.com/), jQuery (https://jquery.com/) and PhoneGap (https://phonegap.com). In addition, the authors report on meta information such as tool licensing, surrounding developer community, and development IDE support. Our study continues where the authors left off, as no empirical results were provided in terms of app and device performance. Additionally, where our study experiment with user interfaces traditionally encountered in regular apps, the artifact developed and evaluated in [22] is in the form of a game, thus inherently requiring animation fluency and API support on a different level than what is required in our implementations and context.

The shortage of studies on cross-platform user experience perspectives is the motivation for de Andrade Cardieri and Zaina’s [23] recent study. They invite end-users and an HCI specialist in an effort to uncover differences in interaction enjoyment between three cross-platform development approaches: Progressive Web Apps, regular mobile websites, and native mobile applications. To the best of our knowledge, this is the first study measuring perception of user interfaces in PWAs compared to native applications. Emotion-based data along with open-ended questions are analyzed and presented. Their findings indicate that participants did not favour the user experience of a specific development approach. This contrasts certain previous studies, including Dalmasso et al.’s comparison of cross-platform tools from 2013, in which the authors describe the quality of user experience in cross-platform apps to be lower than mobile websites and native applications [21]. Cardieri and Zaina’s study could thus indicate that in the years between the studies, user experience in applications developed using cross-platform tools has improved.

However, the user’s perspective of cross-platform approaches is also assessed in a study from 2016 by Mercado et al. [24], in which n=787,228 app user reviews have been classified using Natural Language Processing (NLP). While de Andrade Cardieri and Zaina’s study [23] is of a qualitative and controlled nature, Mercado et al. study the perspectives of performance, reliability, usability and security based on submitted feedback from users in Google’s Play Store and Apple’s App Store. Their results indicate a discrepancy between the iOS and Android platforms, as Android users’ perception of performance, reliability and usability is more negative towards Hybrid applications than native applications. On iOS, Hybrid apps score better on all these concerns, with the exception of performance, i.e., iOS users report of fewer usability-related concerns in Hybrid apps than in Native apps, although this specific Cohen’s *k* model had a relatively low precision compared to the other quality concern models.

Having traversed performance- and user experience-oriented literature, we notice the emergence of certain patterns. In studies scrutinizing the performance of cross-platform generated apps, experiments tend to focus on testing and measuring *device and platform features*, and their effect on hardware and device performance. Examples of such features typically include those of device camera, user’s calendar and contact list, and geolocation and accelerometer [25]. While studies including those discussed throughout this section are of utmost importance to our understanding of limitations, constraints and possibilities posed by cross-platform frameworks, we simultaneously identify a lack of experiments focusing on performance testing graphical user interfaces (GUIs). We find mentions of the importance of user experience and native-like user interfaces to flourish in the literature (e.g., [5,7,26]). While others have focused on the perception of user interfaces, usability and user experience, including Mercado et al. [24] and de Andrade Cardieri and Zaina [23], we target the performance of animated user interfaces on mobile devices, leaving the potential correlation of hardware performance and user perception as a suggestion for further research. According to Noei et al. [27], device attributes including CPU, display and operating system have a significant relationship on user-perceived quality. Drawing from the findings we present in this study, we suggest continuing the examination of user-perceived quality of animations in cross-platform mobile apps, and the correlation with such devices attributes as suggested in [27].

## 3. Research Method

This section is dedicated to presenting our research questions and the research methods employed, thereby the cross-platform frameworks scrutinized, laboratory equipment utilized, and an overview of performance metrics and tools used for measuring and data gathering. We employ a systematic approach for conducting the experiments and gathering data on the performance of the technical artifacts. To ensure verification of results and possibility of re-using the technical artifacts in future research, all developed artifacts have been open sourced and deposited to a Github repository (https://github.com/andreasbhansen/sensors_journal_animations_performance).

### 3.1. Research Questions

Based on our assessment of existing research on performance and user experience in cross-platform development, as presented in Section 2, one overarching objective along with three research questions have been formed. These cover the suitability of metrics included, limitations imposed by profiling tools, and differences in performance between the mobile platforms. We answer the research questions as part of our discussion in Section 6, where each question is re-iterated and answered in a condensed fashion throughout the section’s subsections.

- **Objective:** To investigate the performance of animations and transitions in mobile user interfaces developed using native and cross-platform technologies.
- **Research questions:**
  - **RQ_1_:** Do the included performance metrics fulfil their purpose in researching animated user interfaces in mobile apps?
  - **RQ_2_:** How well do the official performance insight tools cater to the profiling of animation and transition performance in the cross-platform apps developed?
  - **RQ_3_:** Which of the platforms, iOS or Android, required the least amount of device- and hardware resources in order to execute and run performant animations and transitions?

### 3.2. Technologies

To answer our research questions as previously presented, a comprehensive performance scrutiny has been conducted. The outcome of the artifact development is a total of eight mobile apps. The native Android and iOS apps account for two of these, while the three cross-platform frameworks each generate two apps, one for Android and one for iOS, making up for the remaining six apps.

Each framework or technology is also associated with a development approach, as presented in Table 1. Included in the study are three cross-platform approaches, specifically Interpreted, Hybrid and Cross-Compiled. Characteristics of the approaches were previously introduced in Section 1.

The cross-platform frameworks assessed throughout this study were included due to prevalence in either- or both of academia and industry. We find that Xamarin Forms is the most frequently included and scrutinized framework of the Cross-Compiled approach category [28]. Whereas Xamarin has previously seen much scrutiny, React Native and Ionic has not to the same degree enjoyed the focus of researchers. Both these frameworks were included for assessment due to popularity among developers, as reported by the State of JavaScript 2017 survey, exceeding 28,000 responses. The survey illustrate that React Native and Ionic are both popular and well-adopted frameworks within their development approaches [29], respectively Interpreted and Hybrid. While the main survey results do not include any frameworks of the Cross-Compiled approach, we find Xamarin ranking high on a subpage listing “other top mentions” [30], i.e., frameworks that are indeed adopted and in use, but not by enough of the respondents to be placed on the main results page.

The two native apps developed helps us in gathering baseline performance results to accurately understand and measure how the cross-platform apps perform in comparison. Doing any performance- oriented comparisons without the presence of baseline performance results would be inherently challenging. The use of native baselines is also found in other studies, such as in both Ciman and Gaggi’s [16] energy consumption analysis study, and in Willocx et al.’s performance study [20].

It is important to note that no optimization has been done to any of the implementations or code bases. While one could do extensive work in order to optimize performance in all frameworks and technologies included in this experiment, such work is beyond the scope of the study. We are only interested in the performance delivered out of the box, as this is what developers can expect to work with immediately after initializing a new project.

### 3.3. Experimental Setup

The tests were conducted using the mobile devices listed in Table 2. At the time of the experiment, both devices ran the latest operating system updates available. During the tests, both devices were set to maximum screen brightness, had mobile data, GPS and Bluetooth turned off, and did not have any other apps running in the background simultaneously. Each of the apps were restarted prior to each experiment and task, this to help us generate results unaffected by previous experiments.

### 3.4. Metrics and Data Gathering Tools

The following list of metrics have been recorded in this study, deemed of importance to properly scrutinize the performance and possible penalties introduced with the use of cross-platform development frameworks.

#### 3.4.1. Metrics

- **Frames per second (FPS)**: Perceived as an important metric for measuring fluidness of movement in graphical user interfaces [31]. Animations and transitions that are not displayed in a constant close-to-60 FPS can be perceived as *“janky"* according to some studies (e.g., [32]), a term used to describe partially unresponsive interfaces, or interfaces that underperform. Contrastingly, at 60 FPS, an interface and its animated elements is fluently displayed [32]. We test this hypothesis and the importance of measuring FPS throughout our study. During our experiments, we run each animation/transition twice and use the data from the second run, this due to some constraints we found in some frameworks where loading the Lottie animation file contributed to some perceivable lag.
- **CPU usage**: Apps generated in a cross-platform fashion have been reported to differ in terms of CPU usage between the various development frameworks [16,20]. We extend such previous work, and apply methods for measuring CPU usage specifically in the context of animations. We are interested in extending our insight and knowledge in terms of user experience of cross-platform generated apps, and the frequently encountered inclusion of CPU as a mean for measurement makes it ideal to include it here as well, also to discuss the suitability of it in Section 6.
- **Device memory (RAM) usage**: Mobile devices are greatly and inherently constrained by the performance of the hardware they rely on. Especially the availability of memory can hamper the user experience, thus we include a measurement of RAM usage in the tests conducted. We also account for the Lottie animation file (JSON format), which weigh 33KB.
- **GPU memory usage**: We extracted data on GPU memory usage on Android only. We found that on iOS devices, GPU and CPU seem to share the same memory [33], alas, we were unable to identify a method for proper extraction of GPU-only memory usage. As such, Table 3 and Table 4 do not present GPU memory extraction tool or extracted consumption for the iOS platform.

#### 3.4.2. Tools Overview

A handful of tools were selected to measure the four performance-oriented metrics listed previously. The selected tools are listed in Table 3 along with the metrics they assist in extracting. We found that for measuring iOS performance, all but one metric could be measured using *Instruments*, the official profiling tool from Apple. The one metric we could not measure using Instruments was GPU memory usage, as briefly explained in the last section of Section 3.4.1. Measuring Android performance involved the use of both command-line interfaces (CLIs) and a graphical profiling tool, the former being adb (Android Debug Bridge) and the latter being Android Studio, the official development IDE by Google. Android Studio had previously been equipped with a GPU Monitor as part of its array of profiling tools, but it was deprecated and removed from the latest version of the IDE [34]. Thus the need for the CLIs to gather additional insights not provided by Android Studio.

## 4. Artifact Design and Development

This section is dedicated to presenting the three animations/transitions implemented as parts of the artifacts, and an overview of the artifacts’ visual design baseline. To increase the ease of verification of results, we strive to present each animation and transition implementation in a level of detail enabling reproducibility of the artifacts.

### 4.1. Lottie Star Animation

**Figure 1:**
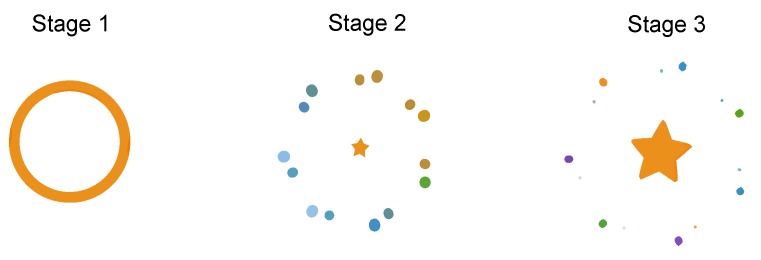
Three primary stages of the Lottie animation.

Lottie is a library developed by the engineering team at Airbnb for rendering of JSON-defined animations exported from Adobe After Effects. The library is cross-platform compatible, and can display animations in both native Android and iOS apps, as well as in Ionic (https://github.com/chenqingspring/ng-lottie), React Native (https://github.com/airbnb/lottie-react-native), Xamarin (https://github.com/martijn00/LottieXamarin) and more. We identified an animation by Michael Harvey on Lottiefiles.com (https://www.lottiefiles.com/72-favourite-app-icon) as the most popular open-source Lottie animation, containing multiple stages and elements, as depicted in Figure 1. The animation involves both movement, appearing and disappearing elements, and is of a fast-paced nature. We deemed this animation interesting for scrutiny purposes due to the variety of elements and its identified popularity as previously mentioned.

### 4.2. Navigation Transition

When navigating between pages and views in mobile apps, transition animations are executed to provide visual indication of an ongoing page (or context) switch. Such transitions vary between platforms and the type of navigation executed, being a modal pop-up, a page switch adding to the navigation stack, a replacement of the current view, and so on. In this paper, we focus on performance testing the transition animation going from one page to another. Inherently, the result from this is coloured by the actual navigation event’s potential performance hit.

The frameworks and native technologies included in this study, as listed in Table 1, did for the most part integrate navigation functionality. Only React Native required a third-party navigation library, and there were several open source alternatives to choose from. While both React Navigation (https://reactnavigation.org/) and React Native Navigation (https://github.com/wix/react-native-navigation) provided easy navigation APIs, the latter was the only library exposing actual native navigation APIs and transitions, not JavaScript-based native-*like* mimics.As such, to make a fair comparison to the other frameworks tested, we opted for navigation using React Native Navigation. For further research, it could be of interest to study the differences between JavaScript-based and native transitions in the context of both device resource usage and user experience.

### 4.3. Side Menu Animation

**Figure 2:**
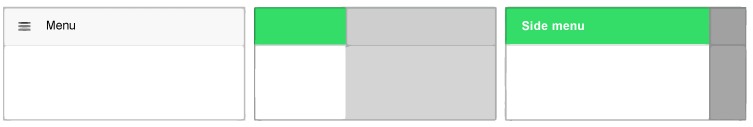
Example of a side-menu drawer opening sequence.

The opening of the side menu and its associated opening animation are typically triggered whenever a user presses the *hamburger menu icon*, i.e., the icon in the very left upper corner in Figure 2, consisting of three stacked horizontal lines. This is a commonly implemented navigation pattern in mobile apps [35].

Achieving the intended behaviour using the frameworks at hand resulted in a variety of implementations, each one seemingly unique to the respective framework, as reported in the following list.

- **Xamarin:** We downloaded an official example code base (https://developer.xamarin.com/samples/xamarin-forms/Navigation/MasterDetailPage/) for implementation of side menus, created by David Britch at Xamarin. Our side menu implementation in Xamarin is based on Britch’s code, with modifications were needed.
- **React Native:** As previously mentioned, we used the React Native Navigation third-party library to enable efficient view navigation. A side menu implementation was also available in the library, thus we decided on using it as the foundation for our implementation.
- **Ionic:** Upon starting a new Ionic project, their CLI prompts with the possibility of scaffolding a side menu-based project, which adds some required and related boilerplate code. We chose this over implementing the same behaviour manually.
- **Native Android:** We found that a side menu-enabled template was available upon project initialization in the Android Studio IDE.
- **Native iOS:** The side menu navigation pattern is not native to the iOS platform, thus no such component existed in the Xcode development environment by default. To compensate for the lack of the component, we found a third-party library which adds an easy-to-use API for providing side menus in Swift iOS, called SideMenu (https://github.com/jonkykong/SideMenu) written by developer Jon Kent.

### 4.4. A Visual Overview

**Figure 3:**
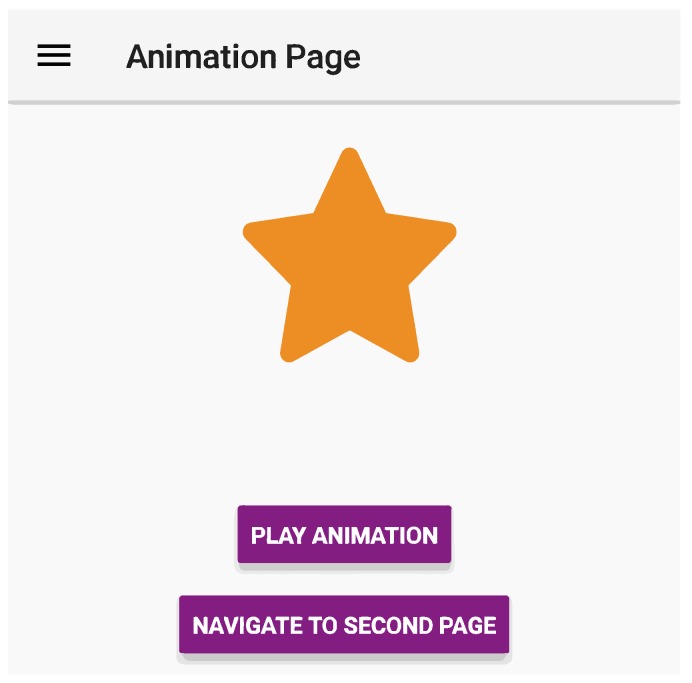
Cropped example of a developed artifact.

Each of the applications developed inherit the same user interface layout and visual design. In Figure 3, the React Native application running on the Android platform is depicted to illustrate the visuals of the finished artifacts. At the top, a navigation bar including an “hamburger menu button” displays the title of the current view. Not depicted is the side menu drawer which will open upon pressing the hamburger menu button, but an example of this is illustrated in Figure 2. Below the navigation bar, following in a vertical stack-wise layout are the animation view (yellow star), a button to execute a play-through of the animation, and at the end a button to navigate to another page.

## 5. Results

The findings from our experiments have been condensed into Table 4, with additional notes on the findings located below the table. For the tasks where the task’s running time could not programmatically be calculated, such as the opening time of the React Native side menu on iOS, we looked to the frameworks’ source code to extract the defined transition or animation duration. We have carefully marked these extracts using an asterisk (*) in the FPS column in Table 4. An example of this is the Side Menu animation on iPhone 6 using React Native, where we had to look to the framework’s source code to find that 250ms was the implemented duration of the animation.

A challenge encountered during the data gathering phase, was the differences in the apps’ performance before conducting the tests. An example of this from Table 4 is the performance of the native Android app, which prior to starting the Lottie animation reported of a memory consumption of 62.34 MB, while the reported consumption was 49.46 MB prior to the navigation transition test. While the differences between these numbers are not massive, they should still be accounted for in research of this nature.

Explanations of the header row and contents of Table 4 follows:

- **FPS**: “*Count^Dur.^ (jank)*” refers to the overall reported *count* of frames rendered, the ***dur**ation* of the animation reported in milliseconds in those cases where duration was less than a full second, and the number of *janky* frames as reported by the Android profiler tool. Take the following example: *18^340 ms^ (2)*, short for 18 FPS rendered in a 340 ms time period, with two frames reported janky. For some of the iOS measurements, we use the hyphen (-) character to report of cross-second FPS results. This is for situations where the iOS Instruments tool’s bar chart visualization of FPS did not report of one single number, but rather two numbers due to spanning more than one second in the visualization. This is further discussed in Section 6.2.2.
- **CPU Peak**: The results of this metric’s measurements are reported in percentage, as they are by the profiling tools on both platforms.
- **Memory Peak**: We report on the performance prior to the animation running *(pre)*, *during* the animation, and after the animation is complete *(post)*. In situations were we could only extract approximate values from the profiling tools, we made use of the tilde ~ character. An example of this is for the Lottie animation on Nexus 5X using React Native, where the *post-animation* memory consumption was approximately 90MB.

### 5.1. Lottie Star Animation Performance

All implementations on Android, including native, reported of large numbers of janky frames during the execution and play-through of the Lottie animation. The native implementation saw the fewest janky frames rendered, used more CPU than React Native and Xamarin, but saw only a minor increase in RAM consumption. For this task, the increase in RAM consumption through all cross-platform frameworks illustrate the penalty introduced with using cross-platform technologies. Ionic’s jump from 93.39 MB to 124.27 MB RAM consumption upon Lottie execution display that there are very much trade-offs to be made when developing cross-platform applications. Another negative aspect of the penalty is how the memory consumption after certain tasks do not decrease to its original value. This is true for all the apps generated using cross-platform technologies.

While Xamarin on Android produced the highest amount of janky frames (more than half of the rendered frames), it had the lowest consumption of CPU power, but consumed the most RAM. On iOS, Xamarin and React Native rendered the highest count of frames. The Xamarin CPU consumption was 30% less than what React Native required, although React Native consumed close to 40 MB less memory.

### 5.2. Navigation Transition Performance

Please do note that we could not isolate the impact caused by the transition animation itself, thus the reported performance is the entirety of the transition, involving the animation and the underlying mechanics for conducting the actual page navigation.

For Android, it is interesting to note how the native app relies the heaviest on the CPU, in fact consuming 9.35% more CPU than the lowest-consuming app being the Xamarin-based implementation, although the latter having a much higher impact on RAM consumption at 108.71 MB versus 73.5 MB for the native app. The highest-consuming implementation in terms of RAM on Android is the Ionic app at 132.76 MB, seeing a major impact upon execution of the navigation transition task, increasing RAM consumption by 41.31 MB. However, Ionic was also the framework to render the most frames of all the Android implementations, although with a high count of reportedly janky frames. It also peaked at a lower CPU percentage than the native app, although 1.78% higher than the Xamarin app.

On the other hand, Ionic on iOS outperform both native and the other cross-platform frameworks on the FPS and CPU usage metrics while consuming more RAM (at 66.65 MB) than native (49.80 mB) and React Native (53.38 MB), but far less than that of the Xamarin implementation at 103.23 MB. Although, one thing to note is how the native app conducts the navigation in 200 ms, whereas the Ionic app to the best of our knowledge, based on Ionic’s source code, uses 500 ms on the same task. Thus, the FPS count is higher for the native app due to the short play-through time, assuming that the length of the navigation is correctly reported.

### 5.3. Side Menu Performance

Again we identify trade-offs in performance, and variations between the platforms. While the Ionic Framework on iOS required the least amount of CPU activity, it rendered only half of the frames compared to the native approach, which used 7% more CPU than Ionic. However, the native approach consumed 26.01 MB less RAM than Ionic. Comparing Android to iOS on side menu navigation, we see that the Ionic Framework consumed 114.79 MB RAM on Android, while 67.14 MB on iOS. These deviations in performance between the platforms result in difficulties when deciding on a specific framework. Another interesting discrepancy is that of native iOS versus native Android, in terms of CPU consumption. In the most extreme case, at 7.94% CPU usage, Android consumed 59.07% less CPU than its iOS counterpart. However, Android still consumed 23.79 MB more memory than the iOS app. This could indicate that variations in performance between platforms is not necessarily caused by cross-platform development frameworks, but are instead an inherent challenge that must be accounted for regardless of development approach.

By comparing the performance of the cross-platform frameworks, we note that React Native on Android rendered the most frames without any reported jank while using only marginally more CPU than the native app and consuming only a few MB more RAM, and using the least amount of GPU RAM. In fact, RAM consumption was only marginally impacted by the execution of the side menu task, which is comparable to that of the native app’s performance. Alas, React Native on iOS did not render an amount of frames comparable to the native app, although using the second most GPU and CPU.

We find that Xamarin on iOS, similar to React Native, render few frames, alas consume more RAM and CPU than the other frameworks. On Android, Xamarin renders consistent frames without jank, at a lower CPU and RAM usage than Ionic. Still, React Native on Android performs better at this task than both Xamarin and Ionic. On iOS, Ionic was the most performant and hardware-consumption friendly framework, rendering more frames than the other cross-platform frameworks, relying less on CPU power than native, and consuming the least amount of RAM compared to the other frameworks.

### 5.4. Additional Observations

Another interesting difference between the platforms is how the performance penalties are clearly observable in the Android sections of Table 4, in which the increase in memory consumption is noted with ease. For the iOS apps, we recorded far fewer drastic increases in hardware penalties. In fact, most of the iOS implementations did not report of any increases in consumption of any of the metrics. This is in stark contrast to Android, where the most significant increase in hardware consumption is that of the Ionic app during navigation transition. In one instance, RAM consumption *decreased* upon execution of the task, being Xamarin during the side menu test, starting at 103.55 MB RAM consumption, decreasing to 87 MB.

## 6. Discussion

Throughout this section, we discuss our findings in the context of the research questions introduced in Section 3.1. Each of the following subsections individually represent one of the research question posed, with additional discussion related to the topic of the question. Also included is a discussion on threats to the validity of our presented results.

### 6.1. Performance Metrics

- Research Question #1: Do the Included Performance Metrics Fulfil Their Purpose in Researching Animated User Interfaces in Mobile Apps?

We did not find the FPS metric to provide much actionable or insightful information. While this metric can be of great value for measuring interface fluidness in games or apps with long-running animations, it did not provide the same value when measuring our fast-paced short-running animations. The other metrics, including CPU, RAM and GPU RAM usage, are common in performance-oriented research (e.g., [13,20,21]). Nevertheless, extraction of GPU memory statistics from the iOS profiling tool was not found to be possible, rendering it less usable for a *cross*-platform comparison. Also, we found that the GPU memory results generated per framework only had minor to no variations between the test, e.g., Ionic which used 4.55 MB regardless of task, or React Native outputting 3.45 MB during the Lottie animation, and otherwise 1.08 MB during the two other tasks. We do not find the GPU memory metric to be of importance for comparative studies on cross-platform development due to the lack of significant differences between the tasks. CPU and RAM metrics were found to provide insightful information on potential performance penalties introduced by cross-platform frameworks.

The difficulty of reasoning about user interface performance based on the FPS metric leads us to believe that user-centric studies on performance and perceived performance is perhaps more generalizable than pure technical empirical assessments. As an example to back this up, as displayed in Table 4, the native Android app rendered only five frames during it’s 328 ms long navigation transition. The reported jank could nevertheless be an indication of unoptimized frame rendering. Further research is needed to find if one can find a correlation between FPS, reported jank and end-users’ performance perception, as our hypothesis is that even the Xamarin Lottie animation reporting 37 janky frames could be difficult to distinguish from the other frameworks reporting of fewer unoptimized frames. This is further elaborated upon in our suggestions for further work in Section 7.1.1.

### 6.2. Evaluation of Performance Profiling Tools

- Research Question #2: How Well do the Official Performance Insight Tools Cater to the Profiling of Animation and Transition Performance in the Cross-Platform Apps Developed?

Both platforms provided their own tooling for performance profiling. We found that in the case of Android profiling, we had to make use of three distinct tools, effectively increasing the overhead of the task. Nevertheless, all the tools employed provided highly granular and understandable data. In the case of the iOS apps, we found the data generated by the profiling tools to be less granular, and no GPU RAM measuring tool was identified. Both platform providers could have created performance profiling experiences requiring less overhead, while providing more data. The Android profiling experience, while requiring several tools and the use of CLIs, outputted the most insightful and actionable data. Below, we discuss in more detail the experiences of gathering data using the profiling tools provided through Android Studio, adb and Xcode’s Instruments.

#### 6.2.1. Android Data Gathering

We did attempt to use third-party programs to measure and gather data on the FPS performance of our artifacts’ user interfaces. GameBench and FPS Meter were frequently mentioned in practitioners’ forums, however both were seemingly unable to properly report on the performance of the implementations, both varying greatly from the results outputted by the official profiling tools, and oftentimes not producing any output at all. Also, as both are apps that are supposed to run on the device in the background while measurements are recorded, we deemed them to have the possibility of negatively impact the performance results.

To retrieve data from the metrics included, a combination of tools was required, as presented previously in Table 3. During the initial data gathering phase, we experimented with a variety of third-party profiling tool alternatives. Due to the experiences from using these tools, we overall avoided the use of them for the data gathering. We found that the app’s performance was impacted by the tools we experimented with. An example of such a tool is the built-in performance monitor in React Native [36], which we found to increase the RAM consumption of the app. In our tests, the initialization and continuous profiling of the React Native monitor increased the RAM consumption from 80.23 MB to approximately 106 MB, with 110 MB at peak consumption during the initializing of the monitor. Such knowledge is invaluable for conducting proper performance testing, as incorrectly reported results could easily be introduced into the data sets if control measurements are ignored.

Another obstacle we encountered while performance testing the applications, specifically measuring CPU usage, was Android 8 (Oreo)’s newly introduced security measures [37], disallowing third-party applications to gain access to CPU data. This limited us to use the official Android Studio system profiler in addition to adb systrace (see Section 6.2.1) for data gathering from the Android-based apps. Thus, we were unable to verify or double-check the results using any third-party software. For the sake of security, this newly implemented measure might have a positive effect, but for the sake of research and system performance insights, it severely limits the possibilities of results verification and ease of access to third-party system monitoring.

On the positive side, we did find the Android Debugging Bridge (adb) to be able to generate highly detailed performance reports using the following command (henceforth referred to as adb systrace ): $ systrace.py –time=5 -o trace_ionic.html sched gfx view -a app_package_name

**Figure 4:**
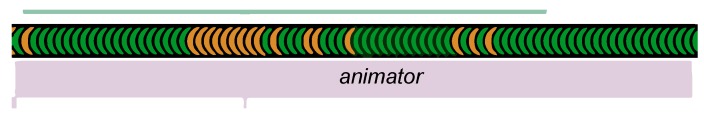
Example of how FPS is reported using systrace for Android apps.

Using the above command, a 5 second snapshot of the specified app’s performance is recorded and outputted in HTML format, which can be opened in a browser and used to visually drill down into single-frame performance issues. Figure 4 illustrates the part of the generated report displaying data on FPS. For the record, every dot seen in the figure represent a single frame, where green ones have been optimally rendered, while orange or red ones represent a frame that has been rendered in a suboptimal fashion. This could be due to the rendering time exceeding 16.6ms per frame, the time available to do any frame-specific calculations and rendering in order to keep a user interface at a stable 60 FPS [38].

For animations and transitions lasting less than a second, being the menu opening animation and the page navigation transition, we have included only the performance measurements reported during that given sequence, and included the measured millisecond count in superscript. For the Lottie animation lasting more than a second, we report on only the first 1000 ms of the animation, being as close to one frame-second as possible.

In terms of retrieval of GPU memory usage statistics, we executed the following terminal command tool: $ adb shell dumpsys gfxinfo app_package_name. The tool was executed directly after each animation event was complete, as the tool outputted data limited to the last 128 rendered frames (note that in periods without changes to the interface, no frames were logged by the tool).

#### 6.2.2. iOS Data Gathering

For the iOS apps, using the official performance profiling tool developed by Apple, Xcode Instruments, granted us access to all the data and metrics needed, with the exception of GPU memory usage. The Instruments tool implement several profiling instruments, several of which reported on slightly similar metrics . We also drew from previous research by Willocx et al. [20] which listed the types of instruments they included, and mapped them to certain metrics. We deviated from some of their suggestions, such as by using the Time Profiler instruments rather than Activity Monitor for CPU measurement, as the reported output rendered results that were easier to interpret. For measuring device memory usage, we employed the VM Tracker Instruments-exposed memory metric Resident Size, described to be the actual device memory consumed by the targeted application [39].

**Figure 5:**
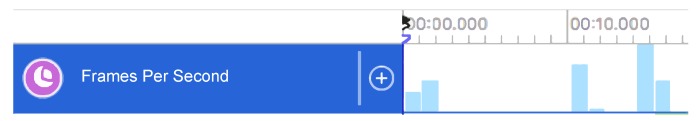
Example of measuring FPS in Xcode’s Core Animations Instruments tool.

Lastly, to measure FPS, we used the Core Animation instrument. Alas, no instruments for reporting on janky frames was identified, rendering investigation of frame-specific problems more difficult than in Android profiling. This resulted in the absence of reporting on janky frames for the iOS implementations. Due to the granularity level and lack of drilling capabilities of the profiling data and instrument, we ended up reporting cross-second FPS counts. This is illustrated in Figure 5, where each bar represents a second of on-screen activity, e.g., playing the Lottie animation. The empty spaces between the bars represent time passed without any frame activity, i.e., no frames were (re-)drawn during those seconds. If an animation is executed well into an already-begun second (Instruments-wise), Instruments will start reporting performance during that second, and finish sometime during the next second slot. Thus, this is the reason for cross-second FPS reporting, in Table 4 illustrated with a dash between the reported FPS, e.g., “30-23” in the iOS native app FPS results during a running Lottie animation.

### 6.3. Platform Performance Deviations

- Research Question #3: Which of the Platforms, IOS or Android, Required the Least Amount of Device- and Hardware Resources in order to Execute and Run Performant Animations and Transitions?

The performance as reported by the profiling tools illustrate an interesting discrepancy, in that the reported CPU usage on Android is consistently lower than that reported on iOS across all three tasks and regardless of technical framework. In fact, CPU usage as reported during the navigation transition task is at 100% on iOS for all but the Hybrid app, a finding correlating to previous research [13] also stating that navigation transition is identified to be more performant in Hybrid apps than in e.g., native due to browser navigation optimization. The only occurrence of both platforms performing similarly, is in the case of the Ionic apps running the Lottie animation, where CPU is peaking at 29.93% on Android, and 30% on iOS.

Nevertheless, it is also important to acknowledge the discrepancy between the native baseline implementations, especially that of CPU consumption during the side menu task, 7.93% on Android versus 67% on iOS. While we could speculate that this is the result of iOS’s lack of a native side menu component (as mentioned, the implementation relied on a third-party component), we do note that iOS in general reports of higher CPU usage, but lower RAM consumption.

To properly answer this section’s research question, one metric is missing from our data set, being that of janky frames in the iOS implementations. We found no suggested approach to extracting this metric from the Instruments profiling tool. Conducting a measurement-based comparison without the presence of this metric is challenging. Nonetheless, we note that the iOS apps are more consistent in the consumption of RAM, while on Android, the apps’ consumption tend to fluctuate.

While there are notable differences between the Android and iOS platforms, the same is also true for the cross-platform frameworks scrutinized. In these last sections of the discussion, we revisit our overarching objective: to investigate the performance of animations and transitions in mobile user interfaces developed using native and cross-platform technologies. While a specific framework can be performant on Android, it is not necessarily the most optimal framework on iOS, and vice versa. An example of this from our study is the cross-platform framework Ionic, which on Android consumes the most RAM in two out of three tests (while also being close to consume the most also in the third), while on iOS it varies between the second- to third most RAM-efficient framework. From previous research, we find similar results in terms of the performance penalty introduced by the WebView component in Hybrid-based apps [7]. Overall, the nature of these platform variations render cross-platform development additionally difficult, as one framework can be most performant on iOS, while on Android a disparate framework can be beneficial to make use of instead. This reaffirms the findings presented by Mercado et al. [24] on differences in perceived performance on Android and iOS. Also, specific product requirements may call for the need of a specific framework, e.g., if Lottie animations are more important than side menu navigation, then one framework may cater better to the requirements than the alternative technologies.

Nevertheless, on Android we deem the Interpreted-based React Native framework to be the most performant cross-platform framework among those included in this experiment. The penalties introduced in terms of hardware consumption are less severe than those caused by Ionic and Xamarin, while also rendering at a higher FPS count than the other frameworks. Also on iOS do React Native produce better results than the alternative frameworks in terms of RAM consumption, but varies between the tasks on CPU usage and consistency of FPS rendered between. In fact, the Hybrid-based Ionic Framework do overall consume between second- and third most RAM, but is the most CPU efficient in two of the tasks, also more so than native, and it renders an FPS that, in two of the tasks, are of an higher count than the other frameworks. The only exception is during the Lottie animation, in which Ionic rendered 11-13 frames less than React Native and Xamarin respectively.

### 6.4. Limitations and Threats to Validity

Due to the vast fragmentation of mobile devices, device types, hardware performance and so on, conducting an experiment of this kind, or verifying the results presented throughout this study, using a different set of devices, could render different results than those we encountered. With more than 24,000 distinct Android devices identified to roam the market [11], research on mobile computing and its subtopics is inherently challenging, and the validity of the research may be limited to devices and device types of similar kind to those included in the experiment. Nevertheless, this is a challenge and practical implication both for research and practice, thus including devices of a certain popularity could increase the validity of the results for the general population. We acknowledge that data from our study is gathered from a limited number of devices n=2. More conclusive results could be gathered by reapplying our research design and re-use of our technical artifacts in a study with a wider array of representative devices, also with multiple devices from the same manufacturer and of the same model to account for any potential differences. Moreover, it could be argued that the FPS metric is more usable when there are different animations running simultaneously or more complex animations shown. Further research should explore the usefulness of FPS as a metric beyond this baseline study.

Another threat to validity is our conscious decision not to conduct any code-wise optimization of the apps to make the animations run smoother or achieve better performance than they would otherwise. As such, artifact implementations developed by expert practitioners with deep understanding and knowledge of platform and framework optimization should be expected to generate different results than those we present. We encourage researchers and practitioners to extend the work presented in this article, and through the optimization of code and architecture attempt to better the performance of animations in cross-platform apps. Nevertheless, the results from our measurements should be the expected performance of a newly initialized project in each of the technologies assessed, executed on the set of devices included.

Differences in feature implementation between the frameworks can also be considered a threat to validity. An example of this is the duration of a transition or animation. For the side menu and navigation transition tasks, each framework implemented the duration of these animations and transitions differently, e.g., navigation in the native Android application had a duration of 328 ms, while Ionic Framework’s duration for the same task was 482 ms. This makes for a difficult comparison between the implementations, an implication worth noting for future studies. The technical artifacts included in this experiment was also developed for the purpose of measuring and extracting data on performance metrics. While a discussion can be had on how well this design may represent real-world complexity, the sheer amount of mobile applications available and the differences between them in terms of design, functionality and complexity would render an attempt to implement generalizable real-world complexity an ambitious task at the very least, if even possible.

There is a continuous push towards improvement of frameworks and technologies by providers of tools and platform. An implication of this is the long-term validity of results and technical artifacts. While this is a threat to the validity of our results, and an inherent limitation to this type of research, this is also equally challenging for practitioners in the industry. An example of this is a recent survey of React Native cross-platform mobile developers, in which “upgrading to a newer version of the framework” is the top-most voted challenge with close to 700 upvotes [40]. Thus, the practical implications of rapid release cycles and continuous advancements in the field is challenging both for industry and research.

## 7. Conclusions and Further Work

While there are manifold studies identified on assessing the performance of cross-platform generated apps, we found a research gap in the lack of performance-oriented evaluations of user interfaces in such apps. Thus, in this study we have focused on empirically scrutinizing the performance of mobile apps developed using cross-platform development frameworks, evaluated in the context of user interface animations and navigation transitions. The frameworks included for assessment in the study is React Native, Ionic Framework and Xamarin Forms. In addition to the cross-platform apps, one native app for each platform, iOS and Android respectively, were also implemented for gathering baseline performance results for comparison purposes.

We also find that the amount of available performance measurement tools, both official and third-party developed, lead to some confusion in terms of picking the right ones, especially as some could, and inherently would, introduce additional overhead in terms of device resource consumption.

The key takeaway from this study is that there are numerous tradeoffs that must be accounted for when choosing cross-platform over native development, especially in terms of memory consumption and differences between the platforms in how performant the frameworks are, but also non-performance benchmarks such as developer proficiency and time-to-market. Specifically, findings from this study indicate that:

- Janky frames were especially prominent in the Lottie animation on Android, in which Xamarin had the highest reported count at 37 sub-optimally rendered frames out of 60 FPS. Performance of the navigation transition and side menu component did not experience suboptimal frame rendering at the same level, although Ionic had the highest count of dropped frames in both tests.
- CPU consumption on iOS was consistently higher than on Android, with an eight-fold difference in the most extreme case between native Android and native iOS in the side-menu test. On iOS, results indicate that Ionic has consistently the lowest CPU consumption across the implementations benchmarked, while it has the highest CPU consumption in two of three tests on Android, specifically Lottie and the side menu tests;
- The impact on memory consumption pre-, during- and post-benchmark varies considerably, especially so between the platforms. In only one case does an iOS implementation consume more memory than it’s Android counterpart, being side menu performance on React Native. Additionally, in only a handful of cases does the memory consumption of an iOS implementation increase during og post benchmark. On Android, we note several cases where the tests’ impact on memory consumption could have practical implications, e.g., Ionic’s 41 MB increase on Android during navigation transition, or native Android’s 24 MB increase during the Lottie animation. Interestingly, in several cases we note that post-benchmark memory consumption does not decrease back to pre-benchmark consumption, possibly indicating memory leaks, slow garbage collection, or similar;
- The variation in GPU memory consumption on the Android platform was small, with React Native and native reporting of the lowest consumptions;
- GPU memory consumption and janky frames could not be measured on iOS using the standard Instruments tool to the best of our knowledge, limiting possibilities for insights and comparison of frameworks and cross-platform approaches on this metric;
- Using FPS as an indication of rendering performance in short-lived animations and transitions in mobile apps proved difficult due to differences between profiling tools on the two platforms. We suggest that combining quantitative performance data, such as FPS, with qualitative data, such as user testing of perceived performance (see Section 7.1.1), could be a more helpful technique to understanding the performance of animations in mobile user interfaces. Nevertheless, quantitative data can still help in identify computing-intensive points in time during the execution of an animation.

There is no silver bullet for mobile app development, neither native nor a specific cross-platform framework has been identified as the unanimously most optimal approach. There are tradeoffs in terms of CPU consumption and memory consumption, where for instance Xamarin on Android has the lowest CPU consumption in the Lottie test, while also consuming the most memory. Increasing the CPU consumption by merely 1.08% will decrease memory consumption by more than 31 MB, which is the case of React Native. Both native and cross-platform frameworks behave differently across the two platforms, certainly so to a degree where choosing the optimal technology across animation tests and metrics is challenging. Additional functional requirements are likely to assist in the decision of a development approach, although this is more a suggestion for further investigation.

### 7.1. Further Work

Two main ideas and topics for further work are presented below, and we encourage researchers to take part in the advancements of empirical knowledge through applied research on cross-platform development, possibly building on the following suggestions.

#### 7.1.1. Performance Versus Perception

While not a topic of direct relevance to the technical nature of this study, it is nevertheless interesting how the author team was unable to distinguish between the Lottie animation running in the native baseline app, versus the same animation running in the Ionic-based Hybrid app during the artifact implementation, regardless of the performance reported in Table 4. Our observations begs the question, how does a system-reported metric such as FPS correlate to an end-user’s actual perception of animation performance? And are the profiling tools developed by the platform providers able to report on issues relating to user perception? Our hypothesis for further work is that for certain types of apps, including those using animations and transitions as means of improving visual aesthetics and user experience rather than for intensive tasks such as device-demanding games, measuring FPS alone is not enough to conclude a user’s experience of an animated interface. In future work, we aim to conduct a study in which end-users are exposed to native and cross-platform apps including animated user interfaces, in order to find if they are able to visually separate the measurement-wise high-performing technologies from the less performing ones.

#### 7.1.2. Continuous Research

Due to the rapid pace at which cross-platform frameworks are released, updated and deprecated, staying updated with the latest of development and technical advances is an inherent challenge to academic research. Nevertheless, new frameworks promise to solve existing challenges, and should thus be assessed and scrutinized accordingly. We suggest to extend this study through inclusion of additional technical frameworks and commercial cross-platform tools, additional to a wider array of test devices, metrics and types of visual animations. The pool of included frameworks could be extended to include such as Fusetools, Flutter and Tabris.js. Also, with the introduction of the Ionic Capacitor tool, enabling the possibility to use any web framework for developing Hybrid apps [41], a new generation of such apps might soon find its way to research and app stores. For web frameworks targeting animation performance as a core focus, perhaps Hybrid apps in the near future will perform even better than the results gathered from our experiments using the Ionic 3 framework. As an extension of our work, custom animations, i.e., no Lottie dependency, could help better communicate the state of the underlying graphic engines provided by each framework. We also encourage future studies to include the frameworks assessed throughout this current experiment, as also they see frequent updates thus possible advances in terms of animation performance.

## Figures and Tables

**Table 1:** Frameworks and Technologies Scrutinized.

Technology	Version #	Approach	Language	IDE
Native Android	26 (SDK)	Native	Kotlin	Android Studio
Native iOS	Xcode 9	Native	Swift	Xcode
React Native	0.49.5	Interpreted	JavaScript	Text editor
Ionic Framework	3.15.2	Hybrid	JavaScript	Text editor
Xamarin Forms	2.4.0.38779	Cross-Compiled	C#	Xamarin Studio

**Table 2:** List of Mobile Devices Included.

Manufacturer	Model	Released	OS (Version)	Memory (RAM)	Processor (CPU)
LG	Nexus 5X	2015	Android (8.0)	2GB	Snapdragon 808
Apple	iPhone 6	2014	iOS (11.1.1)	1GB	Apple A8

**Table 3:** Tools Associated with Metrics.

Platforms	Metrics	Tools
Android	FPS	adb systrace
	RAM	Android Studio Profiler
	CPU %	Android Studio Profiler
	GPU RAM	adb dumpsys
iOS	FPS	Instruments: Core Animation
	RAM	Instruments: VM Tracker
	CPU %	Instruments: Time Profiler
	GPU RAM	-

**Table 4:** Results from Performance Tests.

Technology	FPS *Count^Dur.^* *(jank)*	CPU Peak	Memory Peak (MB) *(pre) during (post)*	GPU Memory *(Android)*
**Lottie Star Animation Performance**				
Nexus 5X (Android)				
Native	60 (16)	25.58%	(62.34) 68.49 (64.14)	2.33 MB
React Native	60 (18)	21.62%	(73.47) 93.72 (∼90)	3.45 MB
Ionic Framework	59 (26)	29.93%	(93.39) 124.27 (∼116)	4.55 MB
Xamarin Forms	60 (37)	19.95%	(116.7) 125.34 (117.08)	6.47 MB
iPhone 6 (iOS)				
Native	30-23	40%	(49.08) 49.08 (49.08)	-
React Native	46-5	100%	(51.96) 53.56 (53.56)	-
Ionic Framework	17-23	30%	(67.75) 67.75 (67.90)	-
Xamarin Forms	48-5	70%	(91.67) 91.67 (101.95)	-
**Navigation Transition Performance**				
Nexus 5X (Android)				
Native	5^328ms^ (3)	29.34%	(49.46) 73.5 (59.06)	2.36MB
React Native	18^340ms^ (2)	22.82%	(68.84) 82.04 (80.40)	1.08 MB
Ionic Framework	21^482ms^ (14)	21.78%	(91.45) 132.76 (104.75)	4.55 MB
Xamarin Forms	18^377ms^ (9)	20%	(105.78) 108.71 (107.21)	6.48 MB
iPhone 6 (iOS)				
Native	18^200ms^	100%	(49.80) 49.80 (49.80)	-
React Native	28^250ms*^	100%	(52.14) 53.38 (53.38)	-
Ionic Framework	36^500ms*^	50%	(66.65) 66.65 (66.65)	-
Xamarin Forms	23-6^586ms^	100%	(103.23) 103.23 (103.23)	-
**Side Menu Performance**				
Nexus 5X (Android)				
Native	27^443ms^ (1)	7.93%	(64.53) 64.92 (64.67)	2.29 MB
React Native	35^573ms^ (0)	11.73%	(69.20) 69.70 (69.60)	1.0 8MB
Ionic Framework	32^617ms^ (8)	21.97%	(93.79) 114.79 (109.84)	4.55 MB
Xamarin Forms	28^458ms^ (0)	13.86%	(103.55) ∼87 (84)	6.36 MB
iPhone 6 (iOS)				
Native	30-^350ms^	67%	(37.63) 41.13 (41.13)	-
React Native	6-4^200ms*^	70%	(76.70) 76.70 (76.70)	-
Ionic Framework	14-8^280ms*^	60%	(67.14) 67.14 (67.14)	-
Xamarin Forms	7-3^300ms*^	75%	(102.45) 102.45 (102.45)	-

## Abbreviations

The following abbreviations are used in this manuscript:

FPS	Frames per second
CPU	Central Processing Unit
RAM	Random Access Memory
GPU	Graphics Processing Unit
CLI	Command-Line Interface
GUI	Graphical User Interface
IDE	Integrated Developer Environment
API	Application Programming Interface
SDK	Software Development Kit
GPS	Global Positioning System
JSON	JavaScript Object Notation
ADB	Android Debug Bridge
HCI	Human-Computer Interaction
NLP	Natural Language Processing
